# “Basal Cell Migration” in Regeneration of the Corneal Wound-Bed

**DOI:** 10.1016/j.stemcr.2018.12.009

**Published:** 2019-01-08

**Authors:** Jan Wijnholds

**Affiliations:** 1 Department of Ophthalmology, Leiden University Medical Center (LUMC), Albinusdreef 2, 2333 ZA Leiden, the Netherlands; 2 Netherlands Institute for Neuroscience, Royal Netherlands Academy of Arts and Sciences (KNAW), 1105 BA Amsterdam, the Netherlands

## Abstract

In this issue of *Stem Cell Reports*, Park et al. (2019) describe real-time *in vivo* visual monitoring of keratin-14^+^, Confetti-labeled limbal epithelial stem cells and their progeny as they contribute to central corneal wound-healing. The authors show that corneal wounds initially heal by “basal epithelial cell migration” into the wound-bed.

## Main Text

The eye contains several tissues and fluidic layers required for clear vision, including the cornea. Cornea tissue has 5 layers: a precorneal tear film adjacent to an epithelium, a thin Bowman’s layer, a broad layer of stroma without blood vessels, a thin layer of Descemet’s membrane, and a single layer of endothelium. The corneal epithelium is stratified, squamous, and non-keratinized and consists of three types of epithelial cells (superficial, wing, and basal). The epithelia secrete peptides with broad anti-bacterial activity. The most apical superficial cells have microvilli and ridges covered by the tear film; they secrete charged glycocalyx, which helps maximize surface area and maintain the tear film. The single layer of epithelial columnar basal cells adheres to the basal lamina adjacent to Bowman’s layer. Adjacent superficial, wing, and basal epithelial cells are held together by desmosomes and the basal cells are anchored by hemidesmosomes to the underlying basal lamina. The epithelia are further anchored by a mesh of collagen plaques and fibrils that interact with the collagen fibrils of Bowman’s layer. The superficial epithelium peels off regularly while basal epithelial cells proliferate and migrate (centripetally) toward the center of the cornea (Hertsenberg and Funderburgh, 2015). But what happens during acute corneal damage? Do the proliferating basal epithelial cells originate from cells close to the wound-bed, or do they originate from the distant peripheral corneal limbus? And which cells are responsible for the regeneration process? Park et al. (2019) address this in this issue of *Stem Cell Reports*.

Acute damage to the corneal epithelial cells can be caused by direct trauma from, for example, chemical burns, microbial invasion, ultraviolet radiation, severe dehydration, or direct physical damage. Fortunately, under normal circumstances, the corneal epithelium can regenerate relatively rapidly after acute damage through a yet-poorly-characterized process of fast-track healing. Still, many patients suffering from severe corneal damage and blurring need corneal transplantations, now routinely practiced clinically for more than 10 years (Rama et al., 2010).

Peripheral to the cornea is the corneal limbus, which is bordered by the conjunctiva. The limbus contains specialized basal columnar epithelial cells adjacent to the other corneal layers of Bowman’s layer, stroma, Descemet’s membrane, and the endothelium. The corneal limbal stroma also contains blood vessels, Langerhans dendritic cells, corneal stromal stem cells, and melanocytes, whereas the corneal stroma itself does not. The limbus is required for proper regeneration of the corneal epithelium after wounding. The specialized epithelial cells in the corneal limbus consist of limbal epithelial stem cells (LESCs) and progeny. Complete removal of the basal limbal epithelium before corneal wounding causes conjunctivalization and neovascularization, which prevents appropriate wound-healing. Wounds heal in the presence of an intact limbal epithelium, and derivative progeny migrate centripetally to regenerate the central corneal epithelium. But what are the exact epithelial cells that migrate centripetally? It is clear that the LESCs are clinically important because loss of limbal stem cells results in conjunctivalization of the cornea, vascularization, inflammation, and loss of corneal transparency. Upon surgical deletion of the LESC pool at the limbus, corneal-committed cells dedifferentiate into bona fide limbal stem cells that retain normal tissue dynamics and marker expression. On the other hand, direct damage to the most peripheral limbal stromal niche abolishes marker expression recovery and leads to pathological wound-healing, indicating that committed corneal cells do possess the plasticity to dedifferentiate, repopulate the stem cell pool, and correctly regenerate the tissue boundary in the presence of intact stroma (Nasser et al., 2018). These data suggested that corneal cells migrate backward to repair the disrupted limbus and then dedifferentiate into functional LESCs.

It is currently thought that following acute wound-healing in the center of the cornea, regeneration takes place through one of two possible processes: the “sliding-cell” or the “rolling-cell” process (Crosson et al., 1986, Kuwabara et al., 1976). The sliding-cell hypothesis suggests that epithelia surrounding the wound move into the damaged area as a block or sheet of cells. The rolling-cell hypothesis suggests that suprabasal epithelia “roll” over leading-edge basal cells to form new leader cells, followed by proliferation, differentiation, and stratification. However, neither model takes into account a clear role for LESCs. Park and colleagues challenge these notions and show by elegant real-time imaging of fluorescently labeled LESCs and derived progeny that corneal epithelia are forced into the wound-bed by an increased population pressure gradient from the limbal epithelia to the wound edge (Park et al., 2019). The authors suggest that central corneal wounds in mice initially heal by “basal cell migration” from the limbus, and they visualize this by real-time imaging of the elevated clonal activity emanating from the limbus, along with basal limbal epithelia, pressed into the wound-bed.

Limbal stem cells are hypothesized to divide either symmetrically, to produce an increased number of new limbal stem cells, or asymmetrically, to produce limbal stem cells that reside in the limbus and transit amplifying cells (TACs) with proliferative potential that move centripetally and replace terminally differentiated cells lost by injury or turnover. However, there is controversy about the direct function of LESCs in wound-healing. The LESCs are slow-cycling stem cells and transplantation of limbal epithelium or corneal epithelium in the mouse cornea with small or no wounds suggested an important role for corneal epithelial cells, but not limbal cells, in the maintenance regeneration of the cornea. In large wounds both the limbal and corneal transplanted epithelia contribute to wound-healing, and these are consistent with the LESC hypothesis and corneal epithelial stem cell hypothesis, respectively (West et al., 2015). But, the true relevance of LESCs in the repair of acute damage to the corneal epithelium was still not clear. Slowly cycling LESCs can however be stimulated to proliferate, so they are likely to play important roles in corneal would-healing. Also, centripetal cell migration is significantly accelerated after removal of dead corneal epithelial tissue. For a long time it was difficult to directly mark the LESC, but in 2015 three groups showed lineage tracing in the adult mouse corneal epithelium (Amitai-Lange et al., 2015, Di Girolamo et al., 2015, Dorà et al., 2015). These data support the LESC hypothesis with quiescent stem cells followed by proliferating stem cells. The K14CreERT2-Confetti mice, which fluorescently label the LESCs and allowed their genetic tracing with up to 10 different colors from stochastic recombination of genes encoding fluorescent proteins, were instrumental in monitoring progenitor cell dynamics within the cornea of living mice. The Confetti mice use the K14 promotor to express tamoxifen-activatable Cre recombinase in K14-positive basal LESCs, which recombined different genes encoding fluorophores in basal LESCs and allowed the centripetal migration of TACs and progeny to be studied. Multicolored corneal limbal epithelium migration could easily be followed in real time *in vivo* from 4 weeks post-tamoxifen application and completed a few months later as a tapered thin layer of cells originating from the limbus extending to the center of the cornea. These authors at that time showed centripetal clonal expansion under homeostatic conditions, suggesting that single LESC progenitor cells are responsible for the production of corneal TAC, and therefore showing that the limbus is a true repository for stem cells (Amitai-Lange et al., 2015, Di Girolamo et al., 2015, Dorà et al., 2015).

But what happens during corneal wound-healing? Can the K14CreERT2-Confetti mice also be used to assess mechanisms of wound-healing in the central cornea? Do the LESCs contribute to the quick initial closure of the central wound-bed, which is far away from the limbus, within 1 to 2 days? Park et al. addressed these questions in corneas *in vivo* in which they created 2 mm central wounds in the epithelial layer; these are relatively large since the diameter of the mouse cornea is only about 2.6 mm. First they showed that 24 hr after injury the central wound showed regeneration with at least one layer of epithelium adjacent to a pronounced new basal membrane and clear signs of stromal inflammation. Stratification of the entire epithelium was restored within 4 weeks of injury. Surprisingly, 24 hr post-injury, the number of BrdU-labeled proliferating basal limbal epithelial cells (LESCs and TACs) in the peripheral limbus increased 3.6-fold. The authors thereafter showed that the regenerated epithelium was indeed derived from K14-positive basal limbal cells, and they confirmed this in flat-mounted whole corneas. A method was then developed to maintain and image corneas in short-term organ culture, to accurately map the spatial-temporal dynamics of K14-positive cells within clones during wound-closure. The data was analyzed by quantitative Spatio-Temporal Image Correlation Spectroscopy. This suggested that basal epithelial cells located close to the wound edge elongated and moved into the damaged area to form a new monolayer of basal epithelial cells. Direction and velocity analysis of clonal migration showed that the overall motion was centripetal, but clones traveled faster at 8 hr than at 36 hr post-injury. *In silico* computational modeling showed that the steeper population-density-driven pressure gradient is sufficient to promote the initiation of centripetal clonal migration during central wound-healing, in agreement with their *in vivo* data. In this way the authors provided direct evidence that basal limbal epithelia are the predominant centripetal cell stream along the basal membrane through population pressure from the increased LESC niche upon central cornea injury. This new basal cell migration hypothesis suggests that peripherally located basal K14-positive LESCs and TACs are activated to proliferate, forcing propagated K14-positive basal TACs into the wound-bed, while synthesizing an immature basal membrane over the first 24 hr (see Figure 7 in Park et al., 2019).

Major questions still remain on the regeneration process, such as, for example, questions on the potential contribution by the stromal inflammatory cells adjacent to the regenerating epithelium. Are these specialized types of inflammatory cells? Do these inflammatory cells secrete signaling molecules that enhance or negatively affect the epithelial wound-healing response? Do the LESCs contribute to the wound-healing process after transplantation of donor cornea tissue or cells? Are there differences in the regeneration process depending on the kind of injury (e.g., bacterial infection of the cornea)? Furthermore, and most importantly, are there differences in regeneration between human and mouse LESCs and TACs? Finally, and potentially importantly for other acute wound-healing studies, the results by Park and colleagues indicate the importance of distant cellular activity to the immediate vicinity of the wound.
